# *δ*-MAPS: from spatio-temporal data to a weighted and lagged network between functional domains

**DOI:** 10.1007/s41109-018-0078-z

**Published:** 2018-07-31

**Authors:** Ilias Fountalis, Constantine Dovrolis, Annalisa Bracco, Bistra Dilkina, Shella Keilholz

**Affiliations:** 1 School of Computer Science, Georgia Tech, Atlanta, USA; 2 School of Earth and Atmospheric Sciences, Georgia Tech, Atlanta, USA; 3 Viterbi School of Engineering, University of Southern California, Los Angeles, USA; 4 Dept. of Biomedical Engr., Georgia Tech and Emory, Atlanta, USA

**Keywords:** Dimensionality reduction, Parcellation, Network inference, Climate teleconnections, Functional brain networks

## Abstract

In real physical systems the underlying spatial components might not have crisp boundaries and their interactions might not be instantaneous. To this end, we propose *δ*-MAPS; a method that identifies spatially contiguous and possibly overlapping components referred to as *domains*, and identifies the lagged functional relationships between them. Informally, a domain is a spatially contiguous region that somehow participates in the same dynamic effect or function. The latter will result in highly correlated temporal activity between grid cells of the same domain. *δ*-MAPS first identifies the epicenters of activity of a domain. Next, it identifies a domain as the maximum possible set of spatially contiguous grid cells that include the detected epicenters and satisfy a homogeneity constraint. After identifying the domains, *δ*-MAPS infers a functional network between them. The proposed network inference method examines the statistical significance of each lagged correlation between two domains, applies a multiple-testing process to control the rate of false positives, infers a range of potential lag values for each edge, and assigns a weight to each edge reflecting the magnitude of interaction between two domains. *δ*-MAPS is related to clustering, multivariate statistical techniques and network community detection. However, as we discuss and also show with synthetic data, it is also significantly different, avoiding many of the known limitations of these methods.

We illustrate the application of *δ*-MAPS on data from two domains: climate science and neuroscience. First, the sea-surface temperature climate network identifies some well-known teleconnections (such as the lagged connection between the El Nin$\tilde {o}$ Southern Oscillation and the Indian Ocean). Second, the analysis of resting state fMRI cortical data confirms the presence of known functional resting state networks (default mode, occipital, motor/somatosensory and auditory), and shows that the cortical network includes a backbone of relatively few regions that are densely interconnected.

## Introduction

Spatio-temporal data become increasingly prevalent and important for both science (e.g., climate, systems neuroscience, seismology) and enterprises (e.g., the analysis of geotagged social media activity). The spatial scale of the available data is often determined by an arbitrary grid, which is typically larger than the true dimensionality of the underlying system. One major task is to identify the distinct semi-autonomous components of this system and to infer their (potentially lagged and weighted) interconnections from the available spatio-temporal data. Traditional dimensionality reduction methods, such as principal component analysis (PCA), independent component analysis (ICA) or clustering, have been successfully used for many years but they have known limitations when the objective is to infer the functional network between all spatial components of the system.

We propose *δ*-MAPS, an inference method that first identifies these spatial components, referred to as “domains”, and then the connections between them (“*δ*-MAPS” section). Informally, a *functional domain* (or simply *domain*) is a spatially contiguous region that somehow participates in the same dynamic effect or function. The exact mechanism that creates this effect or function varies across application domains; however, the key idea is that *the functional relation between the grid cells of domain results in highly correlated temporal activity*. If we accept this premise, it follows that we should be able to identify the “epicenter” or *core of a domain* as a point (or subregion) at which the local homogeneity is maximum across the entire domain. Instead of searching for the discrete boundary of a domain, which may not exist in reality, we compute a domain as the *maximum possible set* of spatially contiguous cells that include the detected core, and that satisfy a homogeneity constraint, expressed in terms of the average pairwise cross-correlation across all cells in the domain. Domains may be spatially overlapping. Also, some cells may not belong to any domain.

After we identify all domains, *δ*-MAPS infers a functional network between them. Different domains may have correlated activity, potentially at a lag, because of direct or indirect interactions. The proposed edge inference method examines the statistical significance of each lagged cross-correlation between two domains, applies a multiple-testing process to control the rate of false positives, infers a range of potential lag values for each edge, and assigns a weight to each edge based on the covariance of the corresponding two domains.

*δ*-MAPS is related to clustering, parcellation (or regionalization), network community detection, multivariate statistical methods for dimensionality reduction such as PCA and ICA, as well as functional network and lag inference methods. However, as we discuss in “Related Work” section and show with synthetic data experiments in “Illustration - Comparisons” section, *δ*-MAPS is also significantly different than all these methods. *δ*-MAPS does not require the number of domains as an input parameter, the resulting domains are spatially contiguous and potentially overlapping, and the inferred connections between domains can be lagged and positively or negatively weighted. Further, the distinction between grid cells that are correlated within the same domain and grid cells that are correlated across two distinct domains allows *δ*-MAPS to separate between local diffusion (or dispersion) phenomena and remote interactions that may be due to underlying structural connections (e.g., a white-matter fiber between two brain regions).

In this paper, we extend (Fountalis et al. 2017) by providing a detailed presentation of the method (including a formal proof of the NP-completeness of the problem) and augment the comparison section (“*δ*-MAPS” section) with more results and methods (e.g., k-means). We proceed with illustrating the application of *δ*-MAPS on data from two domains: climate science (“Application in Climate Science” section) and neuroscience (“Applications in fMRI data” section). First, the sea-surface temperature (SST) climate network identifies some well-known climate “tele-connections” (such as the lagged connection between the El Ni$\tilde {n}$o Southern Oscillation and the Indian ocean) but it also captures less well-known lagged connections that deserve further investigation by the domain experts. Second, the analysis of resting-state fMRI cortical data confirms the presence of three well-known functional brain “networks” (default-mode, occipital, and motor/somatosensory), and shows that the cortical network includes a *backbone* of relatively few regions that are densely interconnected.

## Related Work

A common approach to reduce the dimensionality of spatio-temporal data is to apply PCA (standard or rotated) or ICA techniques. For instance, in climate science, PCA (also known as Empirical Orthogonal Function (EOF) analysis) has been used to identify teleconnections between distinct climate regions (Storch and Zwiers 2001). The orthogonality between PCA components complicates the interpretation of the results making it difficult to identify the distinct underlying modes of variability and to separate their effects, as clearly discussed in (Dommenget and Latif 2002). ICA analysis is more common in the neuroscience literature, aiming to identify independent rather than orthogonal components (Hyvärinen 1999). However, ICA does not provide a relative significance for each component, and the number of independent components should be chosen based on some additional information about the underlying system.

Another broad family of spatio-temporal dimensionality reduction methods is based on unsupervised clustering. Such algorithms can be grouped into region-growing (e.g., (Blumensath et al. 2012; Lu et al. 2003)), spectral (e.g., the NCUT method often applied in fMRI analysis (Craddock et al. 2012; Heuvel et al. 2008) – but also see a discussion of their limitations (Baldassano et al. 2015)), hierarchical (e.g., (Blumensath et al. 2013; Thirion et al. 2014)), probabilistic (e.g., (Baldassano et al. 2015)) or density based methods (Kawale et al. 2013). These groups of algorithms are quite different but they share some common characteristics: the resulting clusters may not be spatially contiguous (Steinbach et al. 2003; Heuvel et al. 2008), every grid cell needs to belong to a cluster (potentially excluding only outliers) (Blumensath et al. 2012; Lu et al. 2003), and the number of clusters is often required as an input parameter (Craddock et al. 2012; Blumensath et al. 2013) - none of these algorithms account for the fact that clusters may overlap. In particular, the lack of spatial contiguity makes it hard to distinguish between correlations due to spatial diffusion (or dispersion) phenomena from correlations that are due to remote (structural) interactions between distinct effects. The proposed method has similar goals (e.g., identification of potentially overlapping spatially contiguous sources of activity) to (Pnevmatikakis et al. 2016) but that method relies mostly on non-negative matrix factorization. Additionally, *δ*-MAPS involves only four hyperparamteters and it is simpler compared to (Pnevmatikakis et al. 2016).

An approach of increasing popularity is to first construct a correlation-based network between individual grid cells, after pruning cross-correlations that are not statistically significant – see (Kramer et al. 2009). Then, some of these methods analyze the (binary or weighted) cell-level network directly based on various centrality metrics, k-core decomposition, spectral analysis, etc. (e.g., (Donges et al. 2009; van den Heuvel and Sporns 2011)) or they first apply a community detection algorithm (potentially able to detect overlapping communities, e.g., (Ahn et al. 2010; Lancichinetti et al. 2011; Palla et al. 2005)) on the cell-level network and then analyze the resulting communities in terms of size, density, location, overlap, etc. (e.g., (McGuire and Nguyen 2014; Power et al. 2011; Steinhaeuser et al. 2010; 2011)). A community however may group together two regions that are, first, not spatially contiguous, and second, different in terms of how they are connected to other regions; an instance of this issue is illustrated in Fig. 4c in the context of climate data analysis.

## *δ*-MAPS

### Background and definitions

The input data is generated from a *spatial field* **X**(*t*) sampled on an arbitrary *gridG*. This grid can be modeled as a planar graph *G*(*V*,*E*), where each vertex in *V* is a grid cell and each edge in *E* represents the spatial adjacency between two neighboring cells. A set of cells *A*⊆*V* is *spatially contiguous*, denoted by *I*_*G*_(*A*)=1, if it forms a connected component in *G*.

The *K-neighborhood* of a cell *i*, denoted by *Γ*_*K*_(*i*), includes *i* and the set of *K* nearest neighbors to *i* according to an appropriate spatial distance metric (e.g., geodesic distance for climate data, Euclidean distance for fMRI data). The *K*-neighborhood of a cell is always spatially contiguous.

Each grid cell *i* is associated with a time series *x*_*i*_(*t*) of length *T* (*t*∈{1,…*T*}). We assume that *x*_*i*_(*t*) is sampled from a stationary signal and denote by $\tilde {\mu }_{i}$ and $\tilde {\sigma }_{i}^{2}$ its sample mean and variance, respectively. The similarity between the activity of two cells *i* and *j* is measured with Pearson’s cross-correlation at zero-lag,

1 $$  r_{i,j} = \frac{\sum_{t=1}^{T} (x_{i}(t)-\tilde{\mu}_{i})(x_{j}(t)-\tilde{\mu}_{j})} {T \, \tilde{\sigma}_{i}\tilde{\sigma}_{j}} \,.  $$

Other similarity metrics could be used instead.

The *local homogeneity at cell i* is defined as the average pairwise cross-correlation between the *K*+1 cells in *Γ*_*K*_(*i*),

2 $$  \hat{r}_{K}(i) = \frac{\sum_{m \neq n\in \Gamma_{K}(i)} r_{m,n}} {K \, (K+1)} \,.  $$

Similarly, we define the *homogeneity of a set of cells A* as the average pairwise cross-correlation between all distinct cells in *A*,

3 $$  \hat{r}(A) = \frac{\sum_{m \neq n\in A} r_{m,n}} {|A|\, (|A|-1)} \,.  $$

### Functional domains

Intuitively, a *domainA* is a spatially contiguous set of cells that somehow participate in the same dynamic effect or function. The exact mechanism that creates this effect or function varies across application domains; however, the key premise is that *the functional relation between the cells of domain A results in highly correlated temporal activity (at zero-lag), and thus high values of the homogeneity metric *$\hat {r}(A)$. A given *homogeneity threshold δ* examines if the homogeneity of *A* is sufficiently high, i.e., a domain *A* must have $\hat {r}(A)>\delta $. (the selection of *δ* is discussed later in this section).

If we accept this premise, it follows that we should be able to identify the “epicenter” or *core of a domain A* as a cell *i*∈*A* at which the local homogeneity $\hat {r}_{K}(i)$ is maximum across all cells in *A* (and certainly larger than *δ*). In general, the core of a domain may not be a unique cell.

More formally now, suppose that we know that cell *c* is in the core of a domain. The *domain A rooted at c* has to satisfy the following three properties: it should include cell *c*, be spatially contiguous, and have higher homogeneity than *δ*:

4 $$  c \in A, \quad I_{G}(A)=1, \quad \hat{r}(A) > \delta \,.  $$

The boundaries of the underlying spatial components of a system are, in practice, unknown and may gradually “fade” into other regions dominated by noise. Instead of trying to identify such “fuzzy boundaries” however, we prefer for simplicity to compute a domain as the *largest possible set of cells* that satisfies the previous three constraints.

**Domain identification problem: Given the field X(*t*) on the spatial grid*G*, a core cell*c*, and the threshold*δ*, the domain*A*(*c*) is a maximum-sized set of cells that satisfies the three constraints of **(4). In Appendix-1 we prove that the decision version of this problem is NP-Hard.

A given spatial field **X**(*t*) may include several domains. The number of identified domains, denoted by *N*, depends on the threshold *δ*. Domains may be spatially overlapping; this is the case when the cells of a region are significantly correlated with two or more distinct domain cores. Also, some cells of the grid may not belong to any domain, meaning that their signal can be thought of as mostly noise (at least for the given value of *δ*). Decreasing *δ* will typically result in a larger number of detected domain cores. Further, as *δ* decreases, the spatial extent of each domain will typically increase, resulting in larger overlaps between nearby domains.

*δ* can simply be a user-specified parameter for the minimum required average cross-correlation within a domain. Another way is to calculate *δ* based on a statistical test for the significance of the observed zero-lag cross-correlations as follows.

We start with a random sample of pairs of grid cells and for each pair *i*,*j* we compute the Pearson correlation *r*_*i*,*j*_ at zero lag. To assess the significance of each correlation we use Bartlett’s formula (Box et al. 2011). Under the null hypothesis of no coupling *r*_*i*,*j*_ should have zero mean, and a reasonable estimate of its variance is given by

5 $$ Var[ r_{i,j} ] = \frac{1}{T} \sum_{\tau_{k} = -T}^{T} r_{i,i} (\tau_{k}) r_{j,j}(\tau_{k})~,  $$

here *r*_*i*,*i*_(*τ*_*k*_) is the autocorrelation of the time series of grid cell *i* at lag *τ*_*k*_. The scaled values $z_{i,j} = \frac { r_{i,j}} { \sqrt { Var[r_{i,j} ]} }$ should approximately follow a standard normal distribution. To assess the significance of each correlation we perform a one sided z-test for a given level of significance *α* (set to 10^−2^ unless specified otherwise).

The threshold *δ* is set as the average of all significant correlations. A domain is a set of spatially contiguous grid cells, thus we require that the mean pairwise correlation for the cells belonging to the same domain to be higher than the mean pair-wise correlation of randomly picked pairs of grid cells. *δ* depends on the choice of the significance level *α*, on the autocorrelation structure of the underlying time series and on the correlation distribution of the field.

#### Algorithm for domain identification

Given the NP-Hardness of the previous problem, we propose a greedy algorithm that runs in two phases. In the first phase, we identify a set of cells, referred to as *seeds*; each seed is a candidate core for a domain. In the second phase, each seed is initially considered as a distinct domain. Then, an iterative and greedy algorithm attempts to identify the largest possible domains that satisfy the three constraints of (4) through a sequence of *expansion* and *merging* operations. The two phases are described next, while the complete pseudocode is presented in Appendix-2. The source code (including supporting documentation) is available online at https://github.com/deltaMAPS/deltaMAPS_fMRI.

**Seed selection** Recall that the core of a domain is a cell of maximum local homogeneity across all cells of that domain. So, one way to detect *potential* core cells, while the domains are still unknown, is to identify points at which the homogeneity field $\hat {r}_{K}(i)$ is locally maximum. Specifically, cell *i* is a seed if $\hat {r}_{K}(i)> \delta $ and $\hat {r}_{K}(i)\geq \hat {r}_{K}(j)$ ∀*j*∈*Γ*_*K*_(*i*). Let *S* be the set of all identified seeds.

In general, a single domain may produce more than one seed because the local homogeneity field can be noisy and so it may include multiple local maxima, greater than *δ*. Further, additional seeds can appear in regions where domains overlap. Consequently, it is necessary to include a merging operation in which two or more seeds are eventually merged into the same domain.

Note that as *K* decreases, the local homogeneity field becomes more noisy and so we may detect more seeds in the same domain. On the other hand, larger values of the neighborhood size *K* can oversmooth the homogeneity field, removing seeds and potentially hiding entire domains. The latter is more likely if the spatial extent of a domain is smaller than *K*+1 cells. This observation implies that the spatial resolution of the given grid sets a lower bound on the size of the functional domains that can be detected.

**Domain-merging operation** Two candidate domains *A* and *B* can be merged if they are spatially contiguous and if the homogeneity of their union is sufficiently high, i.e., $\hat {r}(A\cup B) > \delta $. Whenever there is more than one pair of domains that can be merged, we greedily choose the pair with the maximum union homogeneity; this greedy choice makes the merged domain more likely to expand further.

The merging operation is performed initially on the set of seeds *S*. It is also performed after each domain-expansion operation, whenever it is possible to do so.

**Domain-expansion operation** A domain *A* is expanded by considering all cells that are adjacent to *A*, and selecting the cell *i* that maximizes $\hat {r}(A \cup \{i\})$; again, this greedy choice makes the expanded domain more likely to expand further.

The expansion operation is repeated in rounds. At the start of each round, domains are sorted in decreasing order of homogeneity. Then, each domain is expanded by one cell at a time, as previously described, in that order. After every expansion operation, we check whether one or more merging operations are possible. A round is complete when we have attempted to expand each domain once.

A domain can no longer expand if that would violate the homogeneity constraint *δ* or if there are no other adjacent cells that can be added into the domain. The domain identification algorithm terminates when no further expansion or merging operations are possible.

### The domain network

Given the *N* identified domains *V*_*δ*_={*A*_1_,…*A*_*N*_}, the next step is to construct a network *G*_*δ*_(*V*_*δ*_,*E*_*δ*_) between domains. Different domains may have correlated activity because of direct or indirect interactions. We refer to *G*_*δ*_ as a *functional network* to emphasize that the edges between domains are based on functional activity and correlations instead of structural or physical connections (“structural network”) or causal interactions (“effective network”).

We associate a *domain-level signal* *X*_*A*_(*t*) with each domain *A*. The definition of this signal depends on the specific application field. For instance, when we analyze climate anomaly time series, the domain-level signal is defined as the *cumulative anomaly* across all cells of that domain, where the contribution of each signal is weighted by the relative size of that cell (it depends on the cell’s latitude). For fMRI data, the domain-level signal is defined as the *average BOLD signal* across the cells of that domain.

**Fig. 1:**
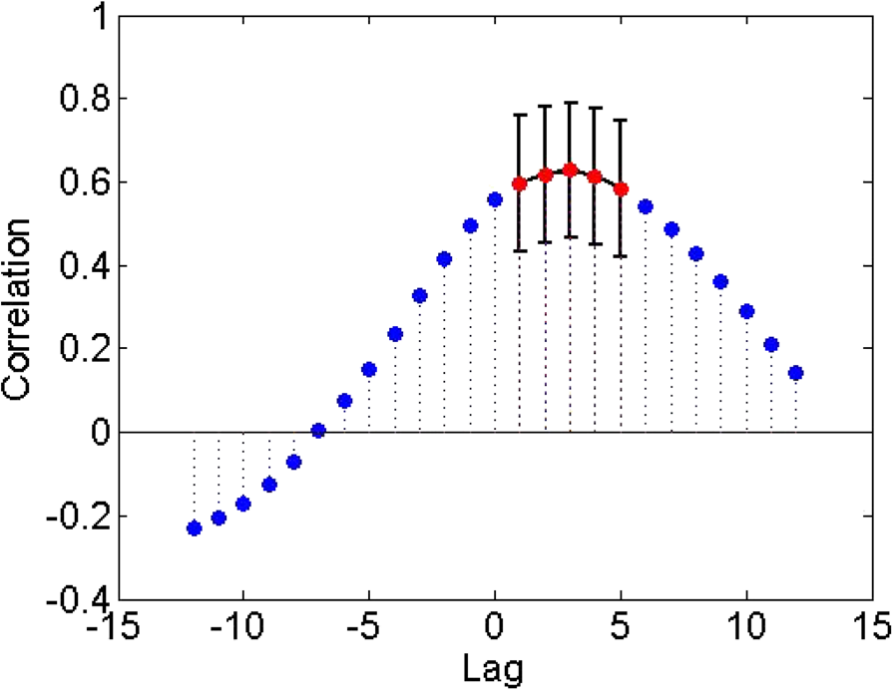
Correlogram between two climate time series for a lag range of ± 12 months. We show the significant correlations for a false discovery rate *q*=10^−3^ with red. The error bars correspond to ± one standard deviation, as estimated by Eq. (7)

Two different domains may be located at some distance, and so they may be correlated at a non-zero lag *τ*. For this reason, we examine if there is a significant cross-correlation between different domains over a range of lags (−*τ*_*max*_≤*τ*≤*τ*_*max*_). The sample cross-correlation between domains *A* and *B* at a lag *τ* can be estimated as:

6 $$  r_{A,B}(\tau) = \frac{ \sum_{t = 1}^{T-\tau} (X_{A}(t) - \tilde{\mu}_{A})(X_{B}(t+\tau) - \tilde{\mu}_{B})} {T \tilde{\sigma}_{A} \tilde{\sigma}_{B}} \,,  $$

where $\tilde {\mu }_{A}$ and $\tilde {\sigma }_{A}$ denote sample mean and standard deviation estimates, respectively. The selection of *τ*_*max*_ should be large enough to include the typical signal propagation delays in the underlying system but at the same time it should be much lower than *T*. The 2*τ*_*max*_+1 cross-correlations for a pair of domains can be represented with a *correlogram*; an example based on climate sea-surface temperature data (see “Application in Climate Science” section) is shown in Fig. 1.

The next step is to examine the statistical significance of the measured cross-correlation between two domains *A* and *B*. Two uncorrelated signals can still produce a considerable sample cross-correlation if they have a strong auto-correlation structure. This is captured by the Bartlett’s formula (Box et al. 2011), which is an estimator for the variance of *r*_*A*,*B*_(*τ*), for a fixed value of *τ* and under the assumption that *A* and *B* are two jointly stationary signals with independent, identically distributed normal errors. Under the null-hypothesis that the domain-level signals of *A* and *B* are uncorrelated,

7 $$ \text{Var}[r_{A,B}(\tau)] = \frac{1}{T-\tau} \sum\limits_{\tau_{k} = -T}^{T} r_{A,A}(\tau_{k}) \, r_{B,B}(\tau_{k}) \,,  $$

where *r*_*A*,*A*_(*τ*_*k*_) is the autocorrelation of the time series of domain *A* at lag *τ*_*k*_.

Under the previous null-hypothesis, the expected value of *r*_*A*,*B*_(*τ*) is zero and the following statistic approximately follows the standard normal distribution *N*(0,1):

8 $$  z_{A,B}(\tau) = \frac{ r_{A,B}(\tau)}{\sqrt{\text{Var}[r_{A,B}(\tau)]}} \,.  $$

The approximation is due to the fact that *r*_*A*,*B*_(*τ*) is bounded between [−1,1]. So, we can now perform hypothesis testing for every pair of domains, computing a corresponding *p*-value based on *z*.

Given that there may be several domains in *G*_*δ*_, we need to control the number of false positive edges that may result from the multiple testing problem. We do so using the False Discovery Rate (FDR) method of Benjamini and Hochberg (1995). Specifically, given *N* domains, we need to perform $M=\frac {N(N-1)}{2}\,(2\tau _{max}+1)$ tests (for each potential edge and for each possible lag value), and compute the *p*-value for each test, based on (8). Given a False Discovery Rate *q* (the expected value of the fraction of tests that are false positives), the Benjamini-Hochberg procedure ranks the *Mp*-values (*p*_*i*_ becomes the *i*’th lowest *p*-value) and only keeps the first *m*<*M* tests (edges), where *p*_*m*_ is the highest *p*-value such that *p*_*m*_<*q* *m*/*M*. 1

**Lag inference and edge directionality** We infer the domain-level network *G*_*δ*_ as follows. Two domains *A*,*B*∈*V*_*δ*_ are connected if there is at least one lag value at which the cross-correlation *r*_*A*,*B*_(*τ*) has passed the FDR test. The standard approach in *lag inference* is to consider the lag value *τ*^∗^ that maximizes the absolute cross-correlation,

9 $$  \tau^{*}_{A,B} = {\arg\max}_{\tau = -\tau_{max} \dots \tau_{max}} \, \{|r_{A,B}(\tau)|\} \,.  $$

The corresponding correlation is denoted as $r^{*}_{A,B}$. There are two problems with this approach. First, it is harder to examine the statistical significance of $|r^{*}_{A,B}|$ because it is the maximum of a set of random variables.^2^ Second, it is often the case that there is a range of lag values that produce “almost maximum” cross-correlations, say within one standard deviation from each other.

Focusing on $\tau ^{*}_{A,B}$ and ignoring the rest of the statistically significant and almost equal cross-correlations is not well justified.

Instead, we follow a more robust approach in which an edge of the domain-level network *G*_*δ*_ may be associated with a range of lag values.^3^ The lag range that we associate with the edge between *A* and *B*, denoted as *R*_*τ*_(*A*,*B*), is defined as *the range of lags that produce significant cross-correlations, within one standard deviation from *$|r^{*}_{A,B}|$. If *R*_*τ*_(*A*,*B*) includes *τ*=0, the edge is represented as *undirected*. If *R*_*τ*_(*A*,*B*) includes only positive lags, the edge is directed from *A* to *B* meaning that *A*’s signal precedes *B*’s by the given lag range; otherwise, we associate the opposite direction with that edge. We emphasize that the directionality of the edges does *not* imply causality; it only refers to temporal ordering.

**Edge weight and domain strength** How to assign a weight to each domain-level edge in *G*_*δ*_? A common approach is to consider the (signed) magnitude of the cross-correlation $r^{*}_{A,B}$. This is reasonable if all domain signals have approximately the same signal power. In addition, we propose a new edge weight that is based on the covariance of the two domains:

10 $$ w(A,B) = \text{cov}[X_{A}(t), X_{B}(t) ] = \tilde{\sigma}_{A} \, \tilde{\sigma}_{B} \, r^{*}_{A,B} \,.  $$

The cross-correlation is computed at lag $\tau ^{*}_{A,B}$ but we could use the average of all cross-correlations in *R*_*τ*_(*A*,*B*) instead. The weight of an edge can be positive or negative depending on the sign of the corresponding cross-correlation.

Finally, the strength of a network node (domain) is defined as the sum of the absolute weights of all edges of that node (ignoring edge directionality).

## Illustration - Comparisons

The two objectives of this section are to illustrate how the *δ*-MAPS method works, and to contrast the results of the latter with commonly used methods such as PCA, ICA, spatial clustering, and overlapping community detection. We rely on synthetic data so that the ground-truth is known.

**Synthetic data description** We construct five domains on a 50 ×70 spatial grid. Each domain *i* is associated with a “mother” time series *y*_*i*_(*t*), (*i*=1 …5). To make the experiment more realistic in terms of autocorrelation structure and marginal distribution, each *y*_*i*_(*t*) is a real fMRI time series with length *T*=1200 (see “Applications in fMRI data” section). The five mother time series *y*_*i*_(*t*) are uncorrelated (absolute cross-correlation < 0.05 at all lags), and they are normalized to zero-mean, unit-variance. To create correlations between domains (i.e., domain-level edges), we construct five new time series *x*_*i*_(*t*) based on linear combinations of two or more mother time series. For instance, if we set *x*_*i*_(*t*)=(1−*α*)*y*_*i*_(*t*)+*αy*_*j*_(*t*+*τ*) with 0<*α*<1 and *x*_*j*_(*t*)=*y*_*j*_(*t*), domains *i* and *j* become positively correlated at a lag *τ*; the correlation increases with *α*. The time series *x*_*i*_ are again normalized to zero-mean, unit-variance. We then scale the time series of domain *i* by a factor $\sqrt {s_{i}}$ to control the variance of each domain (Var[*x*_*i*_(*t*)]=*s*_*i*_).

For simplicity, each domain is a circle with radius *r*_*p*_. A domain has a “core region” with the same center and radius *r*_*c*_<*r*_*p*_; the core is supposed to be the epicenter of that domain. Every point in the core has the same signal *x*_*i*_(*t*) (before we add random noise). Outside the core, the signal attenuates at a distance *d* from the center of the domain as follows:

11 $$ x_{i}(t) = \sqrt{f(d)} \, x_{i}(t), \, f(d)=\frac{r_{p}-d}{r_{p}-r_{c}}, \, r_{c}\leq d \leq r_{p} \,.  $$

Finally, we superimpose white Gaussian noise of zero-mean, unit-variance on the entire grid.

**Fig. 2:**
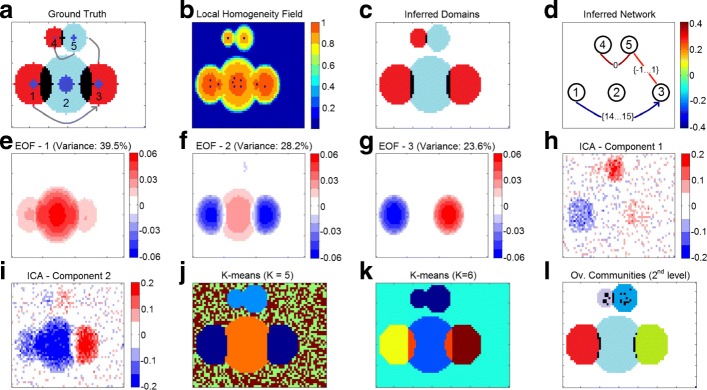
**a** The five ground-truth domains. Adjacent domains have different colors, overlapping regions shown in black, and the core of each domain is in blue. The three constructed edges are shown in gray lines. **b** The homogeneity field $\hat {r}_{K}(i)$ at each cell. The identified seeds are shown in blue. **c** The inferred domains: adjacent domains have different colors and overlaps are shown in black. **d** The inferred domain-level network: the color map refers to the edge correlation. The lag associated with each edge is also shown. **e, f, g** The first three EOF (PCA) components. The variance explained by each component is shown at the top of each figure. **h, i** The two ICA components. **j, k** K-means clustering. **l** The second hierarchical level of community structure as identified by OSLOM: each community has a distinct color and overlaps are shown in black

The parameters of the five synthetic domains are shown in Table 1. The domains differ in terms of size and power (variance). The spatial extent of the domains is shown in Fig. 2a; domains 1 and 3 overlap with domain 2, while domains 4 and 5 also overlap to a smaller extent. Further, there is a strong and lagged anti-correlation between domains 1 and 3, a weaker positive correlation at zero-lag between domains 4 and 5, and an ever weaker positive correlation at zero-lag between domains 3 and 5. The edges of the domain-level network are also shown in Fig. 2a.

**Table 1:** Synthetic area generation parameters

ID	*r*_*c*_	*r*_*p*_	*s*_*i*_	*x*_*i*_(*t*)
1	2	10	16	*x*_1_(*t*)=2/3*y*_1_(*t*)−1/3*y*_3_(*t*+15)
2	4	14	11	*x*_2_(*t*)=*y*_2_(*t*)
3	2	10	16	*x*_3_(*t*)=*y*_3_(*t*)
4	0.5	5	9	*x*_4_(*t*)=3/4*y*_4_(*t*)+1/4*y*_5_(*t*)
5	1	7	6	*x*_5_(*t*)=4/5*y*_5_(*t*)+1/5*y*_3_(*t*)

***δ*-MAPS results** The parameters of *δ*-MAPS are set as follows: *K*=4 cells (up-down-left-right), and *δ*=0.55 (corresponds to significance level 10^−2^). In the edge inference step, the FDR threshold is *q*=10% and *τ*_*max*_=20.

Fig. 2b shows the local homogeneity field $\hat {r}_{K}(i)$ as well as the identified seeds (blue dots), while Fig. 2c shows the five discovered domains. As expected, we often identify more than one seed in the core of each domain due to noise; those seeds are eventually merged into the same domain. The local homogeneity field is weaker in domains 4 and 5 (due to their lower variance) but a seed is still detected in those domains. Seeds also appear at the two overlapping regions between (1,2) and (2,3) but those seeds gradually merge with one of the domains in which they appear.

Each domain is a subset of the domain’s true expanse. The reason is that some cells close to the periphery of each domain have very low signal-to-noise ratio (recall that the signal decays to zero at the periphery and so the average correlation between those cells with the rest of their domain does not exceed the *δ* threshold). More quantitatively, the inferred domains include about 80%-90% of the ground-truth cells in each domain. In non-overlapping regions this fraction is higher (85%-95% of the cells), while in overlapping regions it drops to 45%-80%. The extent of overlapping regions is harder to correctly identify especially when a domain (e.g., domain 2) overlaps with a stronger domain (e.g., domains 1 or 3); the stronger domain effectively masks the signal of the weaker domain. The average pairwise cross-correlation of the cells in each domain varies between 55%-70% in the ground-truth data, while the inferred domains have slightly higher average cross-correlation (65%-75%) due to their smaller expanse.

Finally, Fig. 2d shows the inferred domain-level network. *δ*-MAPS identifies correctly the three edges and their polarity (positive versus negative correlations). The lag ranges always include the correct value (e.g., the edge between domains 1 and 3 has a lag range [14,15]). Also, the three edges are correctly ordered in terms of absolute cross-correlation magnitude: (1,3) followed by (4,5), followed by (3,5).

**PCA/EOF results** PCA assumes that the dominant patterns are orthogonal in space and time (which is not necessarily true, see (Simmons et al. 1983) for a case relevant to climate). To overcome this problem alternative methods exist (e.g., rotated PCA (Vejmelka et al. 2015)) but require more user defined parameters and some times split a single pattern into two different ones (see (Storch and Zwiers 2001; Dommenget and Latif 2002)).

We apply EOF analysis using Matlab’s PCA toolbox. Figure 2e, f, g show the first three principal components, which collectively account for about 90% of the total variance. A first observation is that domains 4 and 5 are not even visible in these components – they only appear in the next two components, which account for about 5% of the variance each. This is because domains 4 and 5 are smaller and have lower variance. This is a general limitation of PCA: the variance of the analyzed field can be dominated by a small number of “modes of variability”, completely masking smaller/weaker regions of interest and their connections. Second, the first three components do not provide a consistent evidence that domains 1 and 3 are strongly anti-correlated; this is due to their lagged correlation, which is missed by PCA. Third, the first component, which accounts for 40% of the total variance, can be misinterpreted to imply that domain 2 is somehow positively correlated with domains 1 and 3, even though it is actually generated by an uncorrelated signal. This is due to the overlap of domain 2 with domains 1 and 3.

**ICA results** We apply ICA on the synthetic data using Matlab’s FastICA toolbox. To help ICA perform better, we specified the right number of independent components, which is two (domains 1,3,4,5 are indirectly correlated – domain 2 is not correlated with any other). The two independent components are shown in Fig. 2h, i. Note that only a rough “shadow” of each domain is visible. Domains 1 and 3 appear in different colors, providing a hint that they are anti-correlated, while domains 3 and 5 appear in the same color because they are positively correlated. Overall, however, the components are quite noisy and it would be hard in practice to discover the functional structure of the underlying system if we did not know the ground-truth. The results are even harder to interpret when we request a larger number of components.

**Clustering results** We apply the most well-known clustering method, *k-means*, on our synthetic data. As commonly done with correlation-based clustering, the distance between two cells *i* and *j* is determined by the maximum absolute correlation across all considered lags, as $1-|r^{*}_{i,j}|$. Figure 2j, k shows the resulting clusters for *k*=5 (the number of synthetic domains) and 6, respectively. For *k*=5, domains 1 and 3 form a single cluster because of their strong anti-correlation; the same happens with domains 4 and 5. The connection between domains 3 and 5 is missed, as well as the overlap between domain 2 with domains 1 and 3. Further, two of the five clusters (green and brown) cover just noise. The situation changes completely when we request *k*=6 clusters. In that case, the overlapping regions in domain 2 form a single cluster, while domains 1 and 3 are separated in different clusters. Another clustering algorithm, resulting in spatially contiguous clusters (Fountalis et al. 2014), is illustrated in “Application in Climate Science” section in the context of climate data analysis (see Fig. 4d).

**Community detection results** We apply a state-of-the-art overlapping community detection method, referred to as OSLOM (Lancichinetti et al. 2011), with the default parameter values. The input to OSLOM is a positively weighted graph: each vertex is a grid cell and an edge between vertices *i* and *j* corresponds to the maximum absolute cross-correlation $|r^{*}_{i,j}|$ across all lags of interest. Absolute correlations less than 30% are considered insignificant and the corresponding edges are pruned.^4^ As most community detection methods, OSLOM does not distinguish between positive and negative correlations. OSLOM provides a hierarchy of communities. When applied to our synthetic data, the first level of hierarchy (not shown) simply groups together domains 1,2,3 in one community (even though domain 2 is uncorrelated with domains 1 and 3), and domains 4,5 in another community. The connection between domains 3 and 5 is missed. The second level of hierarchy is shown in Fig. 2l. Overall, OSLOM does a better job than PCA/ICA/clustering in detecting the spatial extent of each domain. A small overlap between domains (1,2) and (2,3) is discovered but to a smaller extent than *δ*-MAPS. However, a community in OSLOM is not constrained to be spatially contiguous. This is the reason we see some black dots in regions 4 and 5; these are non-contiguous overlaps between the communities that correspond to these two domains.

## Application in Climate Science

We first apply *δ*-MAPS in the context of climate science. Climate scientists are interested in *teleconnections* between different regions, and they often rely on EOF analysis to uncover them (Storch and Zwiers 2001). Here, we analyze the monthly *Sea-Surface Temperature* (SST) field from the HadISST dataset (Rayner et al. 2003), covering 50 years (1956-2005) at a spatial resolution of 2.0^*o*^×2.5^*o*^, and we focus on the latitudinal range of [60^*o*^*S*;60^*o*^*N*] to avoid sea-ice covered regions. Following standard practice, we pre-process the time series to form *anomalies*, i.e., remove the seasonal cycle, remove any long-term trend at each grid-point (using the Theil-Sen estimator), and transform the signal to zero-mean at each grid point.

*δ*-MAPS is applied as follows. We set the local neighborhood to the *K*=4 nearest cells so that we can identify the smallest possible domains at the given spatial resolution. Grid cells that cover mostly land do not have an SST signal and so they do not participate in the local neighborhood *Γ*_*K*_(*i*) of any sea-covered grid cell *i*. Second, the homogeneity threshold *δ* is set to 0.37 (corresponds to a significance level of 10^−2^). In the edge inference stage, the lag range is *τ*_*max*_=12 months (a reasonable value for large-scale changes in atmospheric wave patterns), and the FDR threshold is set to *q*=3% (we identify about 30 edges and so we expect no more than one false positive).

**Fig. 3:**
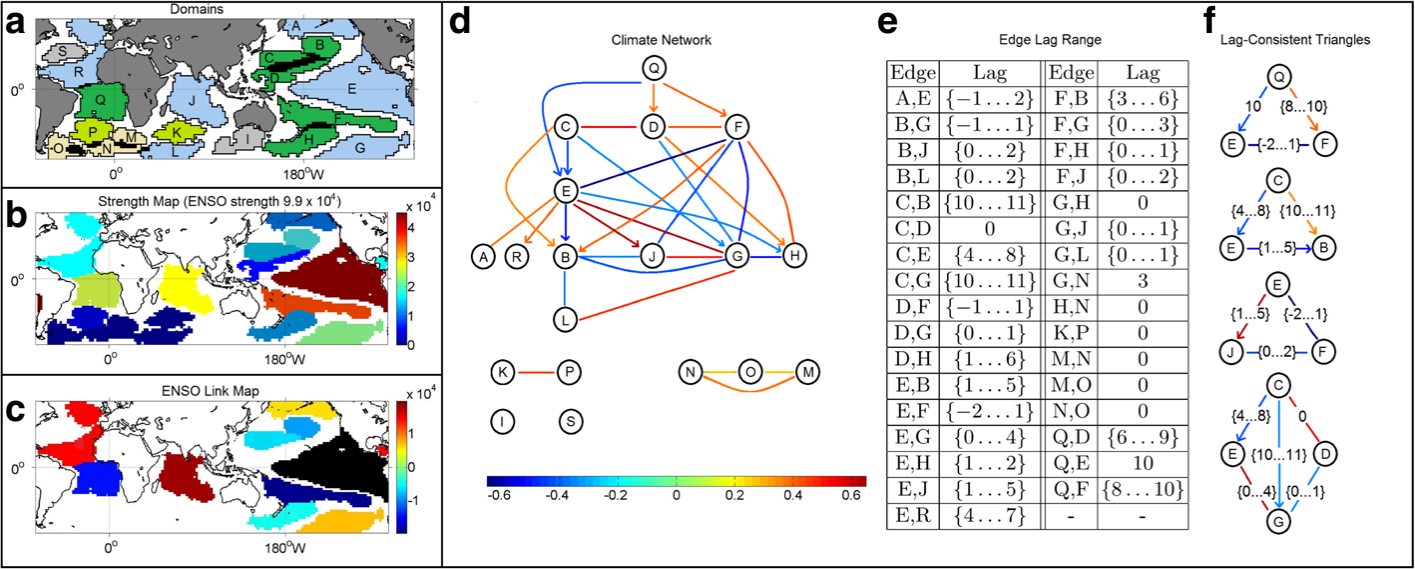
**a** The identified domains. The color of each domain corresponds to the connected component it belongs to (the blue and green nodes belong to two different poles of the same component). **b** Color map for domain strength. The strength of ENSO (domain *E*) is shown at the top. **c** Edges to and from ENSO (shown in black). **d** The climate network. The color of each edge represents the corresponding cross-correlation. **e** The lag range associated with each edge. **f** Examples of lag-constistent triangles

Figure 3a shows the identified domains (the color code will be explained shortly). The spatial dimensionality has been reduced from about 6000 grid cells to 18 domains. 65% of the sea-covered cells belong to at least one domain; the overlapping regions are shown in black and they cover 2% of the grid cells that belong to a domain. The largest domain (domain *E*) corresponds to the El Ni$\tilde {n}$o Southern Oscillation (ENSO), which is also the most important in terms of node strength (see Fig. 3b). Other strong nodes are domain *F* (part of the “horseshoe-pattern” surrounding ENSO), domain *J* (Indian ocean) and domain *Q* (sub-tropical Atlantic). The strength of the edges associated with ENSO are shown in Fig. 3c. These findings are consistent with known facts in climate science regarding ENSO and its positive correlation with the Indian ocean and north tropical Atlantic, and negative correlations with the regions that surround it in the Pacific (horseshoe-pattern) (Klein et al. 1999).

**Fig. 4:**
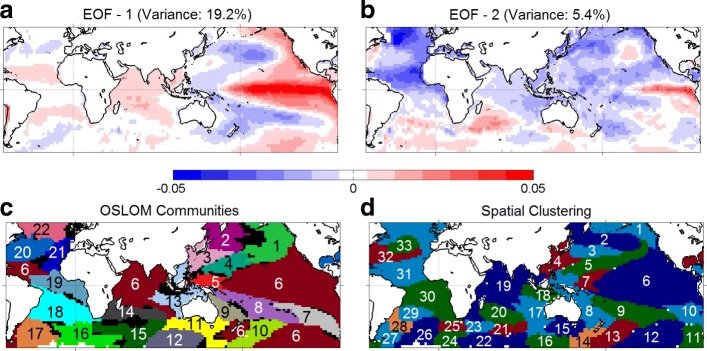
**a**, **b** The first two components of EOF analysis. **c** Communities identified by OSLOM. Each community has a unique number and color. **d** Areas identified by spatial clustering

Figure 3d shows the inferred domain-level network.

The color code represents the (signed) cross-correlation for each edge. The lag range associated with each edge is shown in Fig. 3e; recall that some edges are not directed because their lag range includes *τ*=0. The network consists of five weakly-connected components. If we analyze the largest component (which includes ENSO) as a signed network (i.e., some edges are positive and some negative) we see that it is *structurally balanced* (Easley and Kleinberg 2010). A graph is structurally balanced if it does not contain cycles with an odd number of negative edges.^5^ A structurally balanced network can be partitioned in a “dipole”, so that positive edges only appear within each pole and negative edges appear only between the two poles. In Fig. 3a, the nodes of these two poles are colored as blue and green (the smaller disconnected components are shown in other colors).

Focusing on the lag range of each edge, domain *Q* seems to play a unique role, as it temporally precedes all other domains in the inferred network. Specifically, its activity precedes that of domains *D*, *E* and *F* by about 5-10 months. The lead of south tropical Atlantic SSTs (domain *Q*) on ENSO has recently received significant attention in climate science (Rodríguez-Fonseca et al. 2009; Bracco et al. 2018).

Our results suggest that SST anomalies in domain *Q* may impact a large portion of the climate system. State-of-the-art climate models are generally unable to reproduce the lead relationship between *Q* and the other tropical domains (Barimalala et al. 2012). *δ*-MAPS could be applied across models and different climate fields to identify the origin and impact of this bias (Bracco et al. 2018).

As expected, some of the edges we detect in the network of Fig. 3d are due to indirect correlations. For instance, if *A* has a causal effect on *B* and *C*, at lags *τ*_*A*,*B*_ and *τ*_*A*,*C*_ respectively (suppose that *τ*_*A*,*B*_>*τ*_*A*,*C*_), we may also see a indirect correlation between *B* and *C* at a lag *τ*_*A*,*B*_−*τA*,*C*. Or, it may be that *A* has a causal effect on *B* at lag *τ*_*A*,*B*_ and *B* has a causal effect on *C* at lag *τ*_*B*,*C*_; in that case we may observe an indirect correlation between *A* and *C* at lag *τ*_*A*,*B*_+*τ*_*B*,*C*_. Or, it may be of course that the observed correlation between two nodes *A* and *B* is due to more than one causal paths that originate at *A* and terminate at *B* through one or more nodes.

Switching to lag inference, we say that a triangle is *lag-consistent* if there is at least one value in the lag range associated with each edge that would place the three nodes in a consistent temporal distance with respect to each other. For instance, in the case of the first triangle of Fig. 3f, the triangle is lag-consistent if the edge from *Q* to *F* has a lag of 8 months and the edge between *E* and *F* has lag -2 months (meaning that the direction would be from *F* to *E*); several other values would make this triangle lag-consistent. We have verified the lag-consistency of every triangle in the climate network. One exception is the triangle between domains (*C*,*D*,*G*), shown at the bottom of Fig. 3f. However, the large lag in the edge from *C* to *G* can be explained with the triangle between domains (*C*,*E*,*G*), which is lag-consistent. We emphasize that the temporal ordering that results from these lag relations should not be misinterpreted as causality; we expect that several of the edges we identify are only due to indirect correlations, not associated with a causal interaction between the corresponding two nodes.

For comparison purposes, Fig. 4 shows the results of EOF analysis, community detection, and spatial clustering on the same dataset. The first EOF explains only about 19% of the variance, implying that the SST field is too complex to be understood with only one spatial component. On the other hand, the joint interpretation of multiple EOF components is problematic due to their orthogonal relation (Dommenget and Latif 2002). The anti-correlation between ENSO and the horseshoe-pattern regions is well captured in the first component but several other important connections, such as the negative and lagged relation between the south subtropical Atlantic and ENSO (domains *Q* and *E*, respectively), are missed.

Figure 4c shows the results of the overlapping community detection method OSLOM. Following (Steinhaeuser et al. 2010), the input to OSLOM is a correlation-based cell-level network. Correlations less than 30% are ignored. The weight of each edge is set to the maximum absolute correlation between the corresponding two cells, across all considered lags. OSLOM identifies 22 communities. Community 6 is not spatially contiguous; it covers ENSO, the Indian ocean, a region in the north tropical Atlantic, and a region in south Pacific. This is a general problem with community detection methods: they cannot distinguish high correlations due to a remote connection from correlations due to spatial proximity. In the context of climate, the former may be due to atmospheric waves or large-scale ocean currents while the latter may be due to local circulations.

Finally, Fig. 4d shows the results of a spatial clustering method (Fountalis et al. 2014), with the same homogeneity threshold *δ* we use in *δ*-MAPS. That method ensures that every cluster (referred to as “area”) is spatially contiguous but it also requires that there is no overlap between areas and it attempts to assign each grid cell to an area. Consequently, it results in more areas (compared to the number of domains), some of which are just artifacts of the spatial parcellation process. Further, the spatial expanse of an area constrains the computation of subsequent areas because no overlaps are allowed.

## Applications in fMRI data

Here, we illustrate *δ*-MAPS on cortical *resting-state* fMRI data from a single subject (healthy young male adult, subject-ID: 122620) from the WU-Minn Human Connectome Project (HCP). Our goal is to illustrate that *δ*-MAPS is able to identify well-known resting-state networks even from single subject data, *without having to rely on group-level averages.* The data acquisition parameters are described in (Smith et al. 2013). The spatial resolution is 2mm in each voxel^6^ dimension. The pre-processing of fMRI data requires several steps; we use the “fix-extended” HCP minimal processing pipeline; please see (Glasser et al. 2013). We also perform bandpass filtering in the range 0.01-0.08Hz, as commonly done in resting-state fMRI.

In this paper, we analyze two scanning runs of the same subject (“scan-1” and “scan-2”). Each scan lasts about 14 minutes and results in a time series of length *T*=1200 (repetition time TR=720msec). We emphasize that major differences across different scanning sessions of the same subject are common in fMRI; for this reason, most studies of functional brain networks often only report group-level averages. The entire cortical volume is projected to a surface mesh (Conte69 32K) resulting in about 65K *gray-ordinate* points (as opposed to volumetric voxels). Each point of this mesh is adjacent to six other points; for this reason, we set *K*=6. The homogeneity threshold is set to *δ*=0.37 (inferred using the heuristic proposed in (Fountalis et al. 2014)). The maximum lag range *τ*_*max*_ is set to ± 3, i.e., 2.2 seconds, and the FDR threshold is set to *q*= 10^−4^ (i.e., we expect on average one out of 10K edges to be a false positive).

The application of *δ*-MAPS results in a network with about 850 domains in scan-1 (1120 domains in scan-2). 80% of the domains are smaller than 30-40 voxels (depending on the scan) and 5% of the domains are larger than 250 voxels (Fig. 6a, b shows the identified domains). The number of edges is 4285 in scan-1 (4200 in scan-2). The absolute value of the cross-correlation associated with each edge is typically larger than 0.5. The fraction of negative edge correlations is about 5% in scan-1 and 20% in scan-2 suggesting that the polarity of some network edges may be time-varying. The lag *τ*^∗^ that corresponds to the maximum cross-correlation is 0 in 70% of the edges and ± 1 in almost all other cases. 13% of the edges are directed, meaning that lag-0 does not produce a significant correlation for that pair of domains. There is a positive correlation between the degree of a domain and its physical size (the correlation coefficient between degree and log10(size) is 0.70 for scan-1 and 0.66 for scan-2). Further, the network is assortative meaning that domains tend to connect to other domains of similar degree (assortativity coefficient about 0.7 in both scans).

An important question is whether the *δ*-MAPS networks are consistent with what neuroscientists currently know about resting-state activity in the brain. During rest, certain cortical regions that are collectively referred to as the *Default-Mode Network (or DMN)* are persistently active across age and gender (Yeo et al. 2011). Other known Resting-State Networks (RSNs) are the occipital (part of the visual system) and the motor/somatosensory (associated with planning and execution of voluntary body motion). With the terminology of network theory, the previous “networks” would be referred to as *communities* within the larger functional brain network. To identify communities in the *δ*-MAPS network, we applied OSLOM (Lancichinetti et al. 2011). OSLOM identifies two hierarchical levels in both scans. The first level consists of highly overlapping communities that cover almost the entire cortex. The second hierarchical level is more interesting, resulting in eight communities for scan-1 (nine for scan-2). Figure 6e, f shows the three communities (C.1, C.2, C.3) for each scan that have the highest resemblance to the three previously mentioned resting-state networks: C.1 corresponds to the DMN, C.2 corresponds to the occipital resting-state network, and C.3 corresponds to the motor/somatosensory network. C.1 is quite similar across the two scanning sessions and it clearly captures the DMN. In C.2, the extent of the network is smaller in scan-2, which is not too surprising giving the known inter-scan variability of resting-state fMRI. C.3 is also quite similar across the two scans and consistent with the motor/somatosensory network.

**Fig. 5:**
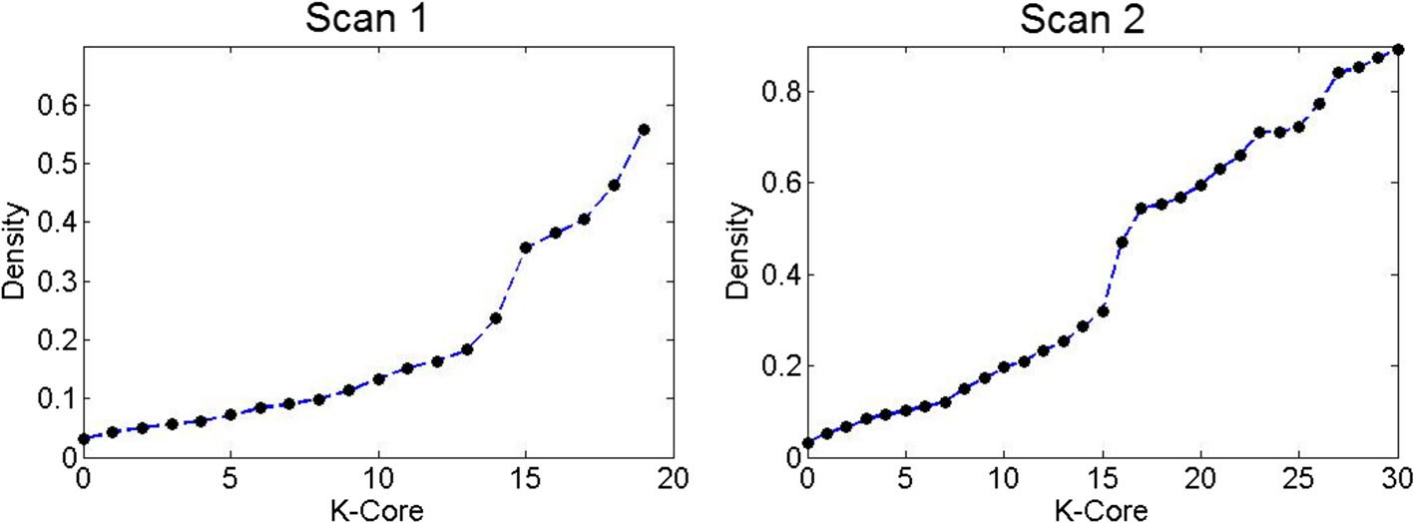
Network density as a function of the k^*th*^ core for scan-1 (left panel) and scan-2 (right panel)

To further investigate the structure of those higher degree (and typically larger) domains, we perform *k-core decomposition* (Alvarez-Hamelin et al. 2006). The k-core decomposition process starts with the original network (*k*=0), and it removes iteratively all nodes of degree *k* or less in each round so that after the extraction of the *k*’th core all remaining nodes have degree larger than *k*. The density of the remaining network, as shown in Fig. 5, after the extraction of *k*=14 cores from the scan-1 network (*k*=16 cores in scan-2) shows a sudden increase by a factor of two. This suggests that the network includes a *densely inter-connected backbone*. The size of this backbone is small relative to the entire network: 130 domains in scan-1 (90 in scan-2).

**Fig. 6:**
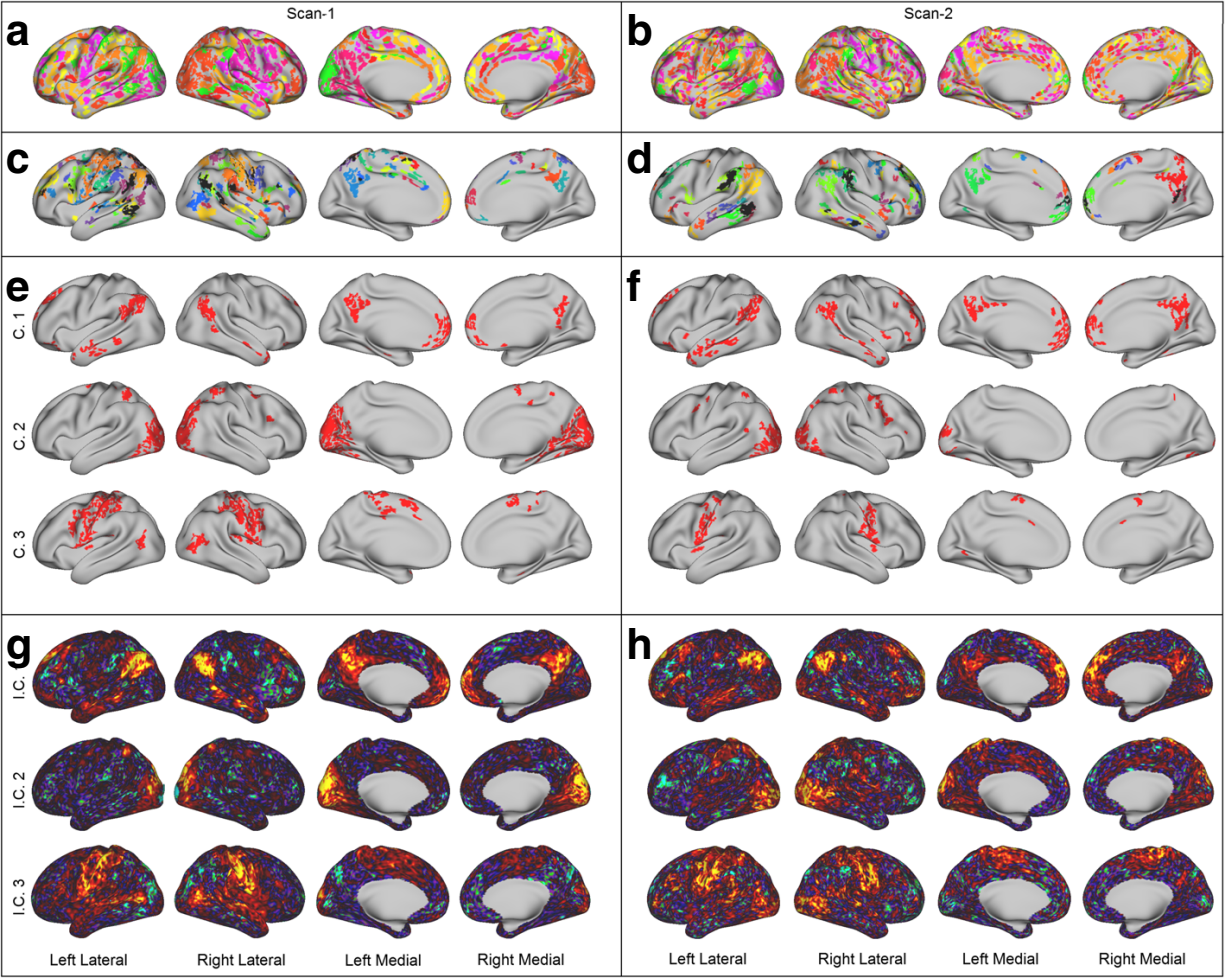
Results for scan-1 (left panel) and scan-2 (right panel). (**a**, **b**) The identified domains, each domain is assigned a color randomly, overlaps are shown in green. (**c**, **d**) The domains of the backbone network, each domain is assigned a color randomly, overlaps are shown in black. (**e**, **f**) Three domain-level network communities for each scan. The first corresponds to the default-mode network, the second to the occipital network, and the third to the motor/somatosensory network. (**g**, **h**) Three independent components (corresponding to the domain level communities in (**e**, **f**)) as identified by MELODIC ICA

Similar observations about resting-state functional brain networks have been previously reported based on a rich-club network analysis method (van den Heuvel and Sporns 2011). Fig. 6c, d shows the location of the backbone domains for each hemisphere and for each scan. The regions that are usually associated with the DMN dominate the backbone of both sessions. Interestingly though, scan-1 includes the regions of the motor/somatosensory network, while the backbone of scan-2 is missing those regions.

We conclude the analysis comparing our results to those obtained by ICA. ICA, in contrast to the proposed method, aims to identify temporally coherent components (ignoring the need for spatial contiguity). Here, we use MELODIC ICA (Beckmann and Smith 2004) to identify 7 independent components (ICs) for each scan^7^; here we only show the components that are more similar to the RNNs presented in Fig. 6e, f. The three ICs correspond to the DMN (I.C. 1), occipital (I.C. 2), and motor/somatosensory (I.C. 3) network. We also observe differences in terms of activation strength between the two scans. Interestingly, these differences are “reflected” in both methods. Compare for example the size of the identified communities in the occipital network to the strength of the activations in the corresponding IC. The similarity of results from two qualitatively different methods, encourages us to believe that *δ*-MAPS can identify meaningful functional components and infer their connectivity.

## Discussion

In climate science future possible applications of *δ*-MAPS range from quantifying uncertainties in climate projections to diagnosing changes in teleconnections in response to anthropogenic perturbations. Furthermore *δ*-MAPS can be successfully applied towards quantifying differences across datasets and models, evaluating model performances, and investigating model biases and their propagation across different fields of the climate system. A more in-depth discussion of these aspects and applications can be found in (Bracco et al. 2018).

*δ*-MAPS results in a correlation-based functional network. A next step would be to infer a causal, or *effective* network, leveraging the framework of probabilistic graphical models. For example, in (Ebert-Uphoff and Deng 2014) the authors leverage probabilistic graphical models to construct a causal climate network using pre-defined climate indices. Instead of attempting to construct a graph in which the nodes are arbitrarily defined, one could leverage *δ*-MAPS to identify the underlying structure and then apply conditional independence tests to remove non-causal edges. Further, probabilistic graphical models always result in a directed acyclic graph (DAG). However, in many cases (e.g., climate) feedback loops exist, thus such a framework is not a realistic model for the system’s dynamics. Alternative approaches to establish causal inference could be based on Granger causality or controlled interventions (Hlinka et al. 2013; Holland et al. 1985).

Additionally, in many real systems the underlying temporal dynamics are non-stationary. Instead of relying on sliding window-based approaches, which are often sensitive to the duration of the window, an important extension of *δ*-MAPS will be to construct dynamic networks by detecting automatically the time periods during which the network remains constant. It would also be interesting to combine the inferred functional network with a structural network that shows the physical connectivity between the identified domains. This is not hard in the case of communication networks but it becomes also feasible for brain networks using diffusion-weighted MRI. The projection of the observed dynamics on the underlying structure can help to characterize the actual function and delay of each system component. Finally, here we assume that a *universal* threshold *δ* can be applied across the spatial extent of the spatio-temporal field. However, an alternative would be to apply different thresholds for different regions of the field.

## Conclusions

In this paper we present *δ*-MAPS, a method suitable for the analysis of spatio-temporal data. At a first step, *δ* maps identifies “domains”; the functional components of a spatio-temporal system. *δ*-MAPS is based on the premise that the functional relation between the grid cells of a domain results in highly correlated temporal activity. To this end it first identifies the “epicenter” or “core” of a domain as a point (or set of points) where the local homogeneity is maximum across the entire domain. Instead of searching for the discrete boundary of a domain, which may not exist in reality, we compute a domain as the *maximum possible set* of spatially contiguous cells that include the detected core, and that satisfy a homogeneity constraint *δ*.

At a second step, *δ*-MAPS infers a functional network between domains. Different domains may have correlated activity, potentially at a lag, because of direct or indirect interactions. The proposed network inference method examines the statistical significance of each lagged cross-correlation between two domains, applies a multiple-testing process to control the rate of false positives, infers a range of potential lag values for each edge, and assigns a weight to each edge based on the covariance of the corresponding two domains.

Using *δ*-MAPS we analyzed the temporal relationships between different functional components of the climate system in the sea surface temperature field. We found that the proposed method successfully uncovered many well-known climate teleconnections and the lag associated with them. In the context of neuroscience, we performed a single subject analysis focusing on resting state fMRI data. We found that the proposed method was able to uncover many of the well-known resting state networks. We also show how the method identifies a small number of strongly interconnected areas forming the backbone of the resting state network. Finally, using synthetic data we also show how *δ*-MAPS overcomes limitations of traditional dimensionality reduction techniques such as PCA/ICA, clustering and overlapping community detection.

## Appendix 1: Identifying the largest domain is NP-complete

We are given a spatio-temporal field **X**(*t*) on a grid *G*, a pairwise similarity metric between pairs of grid cells and a threshold *δ*. Starting from a grid cell *c*, the goal is to find the largest subset of grid cells that form a single spatially connected component, and whose average similarity exceeds the threshold *δ*. The spatial grid can be represented as a planar graph *G*(*V*,*E*) where each grid cell is a node and edges connect adjacent grid cells. Formally we have the following graph optimization problem:

*Definition 1.* Rooted Largest Connected *δ*-Dense Subgraph Problem (rooted LC *δ*DS). Given a regular (grid) graph *G*(*V*,*E*), a weight function $w: V\times V \rightarrow \mathbb {R}$ (where *w*(*v*,*v*)=0 and symmetric), a threshold *δ*, and a node *c*∈*V*, find a maximum cardinality set of nodes *A*⊆*V* such that *c*∈*A*, the induced subgraph is connected (*I*_*G*_(*A*)=1) and $\frac {\sum _{v,u\in A}w(v,u)}{|A|(|A|-1)} > \delta $ (i.e., $\hat {r}(A) > \delta $).

To show that rooted LC *δ*DS is NP-hard we first consider a variant of the problem in which the induced subgraph *A* has to satisfy two conditions; it has to be a connected subgraph of *G*, and the average weight of the edges in *A* has to exceed *δ*. More formally:

Definition 2. Largest Connected *δ*-Dense Subgraph Problem (LC *δ*DS). Given a regular (grid) graph *G*(*V*,*E*), a weight function $w: V\times V \rightarrow \mathbb {R}$ (where *w*(*v*,*v*)=0 and symmetric), and a threshold *δ*, find a maximum cardinality set of nodes *A*⊆*V* such that *I*_*G*_(*A*)=1 and $\hat {r}(A) > \delta $.

To show that LC *δ*DS is NP-hard we use a reduction of the densest connected *k* subgraph problem.

Definition 3. Densest Connected *k*-Subgraph Problem (DC*k*S). Decision version: Given a graph *G*(*V*,*E*), and positive integers *k* and *j*, does there exist an induced subgraph on *k* vertices such that this subgraph has at least *j* edges and is connected?

DCkS (also referred to as the connected h-clustering problem) has been shown to be NP-complete on general graphs (Corneil and Perl 1984), as well as on planar graphs (Keil and Brecht 1991). DC*k*S is polynomially time solvable for subclasses of planar graphs of bounded tree width (Arnborg and Seese 1991). Grid graphs, which are the type of graphs that arise in our application domains, are planar bipartite graphs, with non-fixed tree width, and no positive results are known for this subclass of planar graphs. The work on approximating densest/heaviest connected k-subgraphs is relatively very limited (see recent theoretical result (Chen et al. 2015)). It is easy to show that the DC*k*S problem can be easily reduced to an instance of the decision version of the LC *δ*DS problem, and hence it is also NP-complete even on planar graphs.

LEMMA 1. The decision version of the LC *δ*DS problem is NP-complete on planar graphs.

PROOF. This can be shown via a reduction from the DC*k*S. We reduce an instance <*G*,*k*,*j*> of the DC*k*S to an LC *δ*DS instance by using the same graph *G*, setting *w*(*u*,*v*)=*I*(*u*,*v*)∈*E* (*w*(*u*,*v*) is 1 if and only if the pair of nodes is connected by an edge), and *δ*=*j*/*k*(*k*−1).

Now it is easy to show that rooted LC *δ*DS is also NP-hard. If a poly-time algorithm existed for the rooted LC *δ*DS, then by calling it |*V*| times with each of the nodes of the graph, we would obtain in poly-time a solution to the NP-hard LC *δ*DS.

## Appendix 2: *δ*-MAPS pseudocode


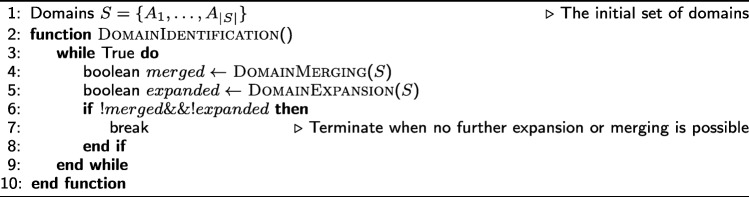



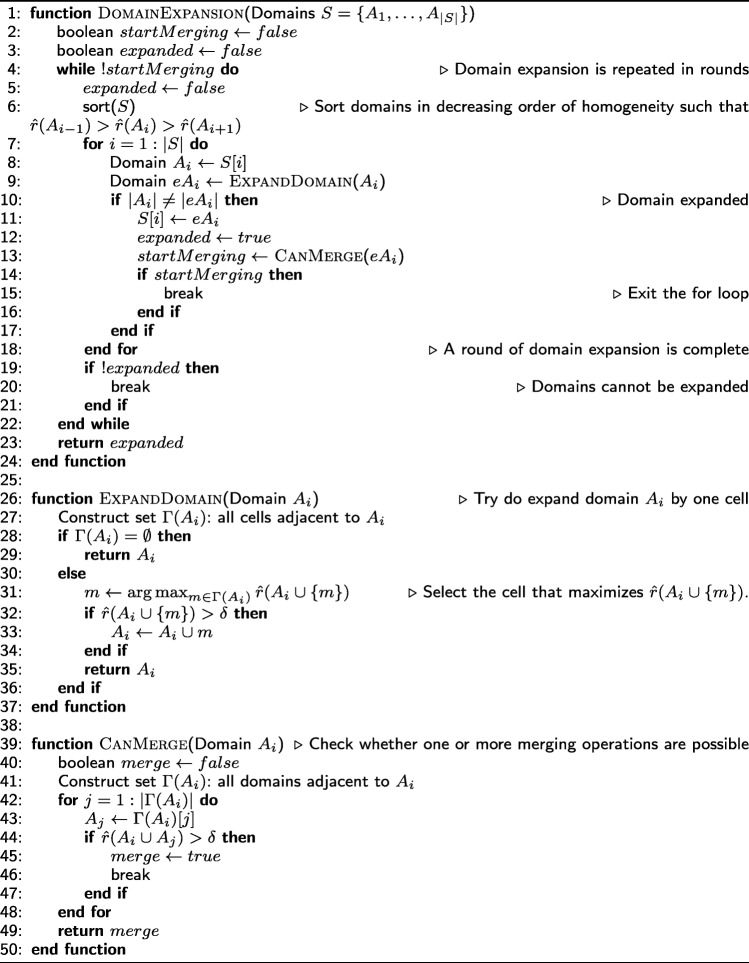



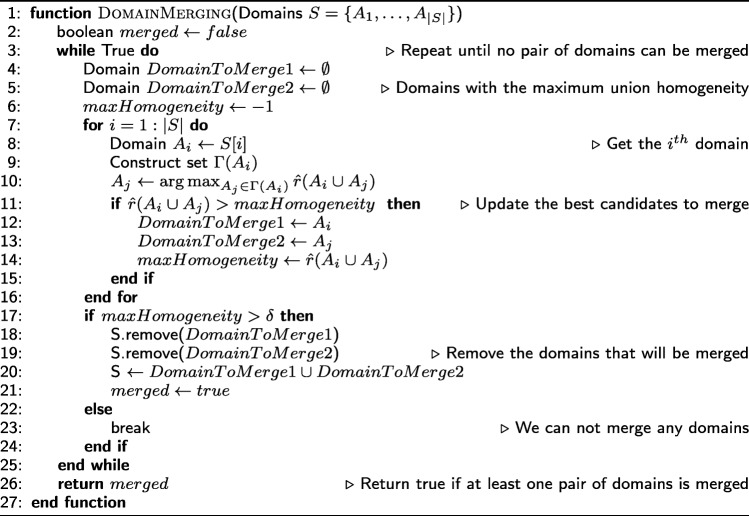


## Abbreviations

DAG: Directed Acyclic Graph
DCkS: Densest Connected *k*-Subgraph Problem
DMN: Default Mode Network
ENSO: El Ni$\tilde {n}$
EOF: Empirical Orthogonal Functions
FDR: False Discovery Rate
fMRI: Functional Magnetic Resonance Imaging
HCP: Cumman Connectome Project
ICA: Independent Component Analysis
LC *δ*DS: Largest Connected *δ*-Dense Subgraph Problem
PCA: Principal Component Analysis
RSN: Resting State Network
SST: Sea Surface Temperature

## Footnotes

1 This formula assumes that the *p*-values are independent (which is often not true in practice). The case of correlated p-values can be handled replacing *q* qith $q/\sum _{i=1}^{m} 1/i$, but that approach is very conservative, resulting in many false negatives (Reiner et al. 2003).

2 An analytic approach based on extreme-value statistics was proposed in (Kramer et al. 2009) but it relies on several approximations. Numerical approaches based on frequency-domain bootstrapping, on the other hand, are computationally expensive (Kramer et al. 2009; Martin and Davidsen 2014; Rummel et al. 2010).

3 In principle, it may be a set of lag values. In practice though, significant correlations result for a continuous range of lag values.

4 We have experimented with other pruning thresholds between 20%-50% and the results are very similar at the first two hierarchy levels.

5 For instance, if two friends are both enemies with a third person, they form a balanced social triangle.

6 Grid cells are referred to as voxels in the fMRI literature.

7 MELODIC ICA has an option to automatically estimate the number of ICs to return. Choosing this option yielded approximately 200−250 components in each scan. Activations were much lower than the ones shown in Fig. 6g, h both in strength and spatial extent. We could not identify RSNs similar to those shown here.
